# Nanobodies targeting SARS-CoV-2 papain-like protease exert dual antiviral and anti-inflammatory effects

**DOI:** 10.1128/jvi.00599-26

**Published:** 2026-08-28

**Authors:** Guolong Liu, Jiantao Chen, Fang Wu, Peilan Wei, Yuzhe Wu, Di Yan, Xinfeng Xu, Qiang Ding, Yanqun Wang, Shuwen Liu, Jincun Zhao, Wei Xu

**Affiliations:** 1 Guangdong Provincial Key Laboratory of New Drug Screening, School of Pharmaceutical Sciences, Southern Medical University 608582, Guangzhou, China; 2 NMPA Key Laboratory for Research and Evaluation of Drug Metabolism, School of Pharmaceutical Sciences, Southern Medical University 608582, Guangzhou, China; 3 Guangdong-Hong Kong-Macao Joint Laboratory for New Drug Screening, School of Pharmaceutical Science, Southern Medical University, Guangzhou, China; 4 Guangdong Provincial Center for Disease Control and Prevention, Chinese Academy of Medical Sciences 624661, Guangzhou, China; 5 Guangdong Provincial Key Laboratory of Pathogen Detection for Emerging Infectious Disease Response, Guangdong Provincial Center for Disease Control and Prevention 278599 , Guangzhou, China; 6 Guangdong Workstation for Emerging Infectious Disease Control and Prevention, Chinese Academy of Medical Sciences, Guangzhou, China; 7 State Key Laboratory of Respiratory Disease, Guangzhou Institute of Respiratory Health, The First Affiliated Hospital of Guangzhou Medical University 664066, Guangzhou, Guangdong, China; 8 National Clinical Research Center for Respiratory Disease, Guangzhou Institute of Respiratory Health, The First Affiliated Hospital of Guangzhou Medical University, Guangdong, Guangzhou, China; 9 Center for Infection Biology, School of Basic Medical Sciences, Tsinghua University 12442, Beijing, China; 10 State Key Laboratory of Multi-organ Injury Prevention and Treatment, Southern Medical University 70570, Guangzhou, China; 11 MOE Key Laboratory of Infectious Diseases Research in South China, Southern Medical University 70570, Guangzhou, China; 12 MOE Innovation Center for Medical Basic Research on Inflammation and Immune Related Diseases, Southern Medical University 70570, Guangzhou, China; University of Michigan Medical School, Ann Arbor, Michigan, USA

**Keywords:** nanobodies, SARS-CoV-2, PLpro, antiviral activity, PLpro-induced intestinal inflammation

## Abstract

The emergence of SARS-CoV-2 variants and the rise of antiviral resistance necessitate the development of novel therapeutics targeting highly conserved viral proteins. The papain-like protease (PLpro) is a critical target due to its dual role in viral replication and immune evasion, particularly through the removal of ISG15 modifications from host proteins. However, nanobody-based strategies targeting PLpro for combined antiviral and anti-inflammatory purposes remain underdeveloped. This study reports the identification of two nanobodies, NbP1 and NbP2, that specifically disrupt the PLpro/ISG15 interaction interface. We characterized their binding specificity and affinity using yeast surface display and competitive fluorescence polarization assays. To ensure intracellular efficacy, the nanobodies were conjugated with cell-penetrating peptides (CPPs), resulting in significant inhibition of viral replication and the attenuation of inflammatory cytokine responses in both human colonic cells and a murine colitis model. Structural epitope mapping revealed that both nanobodies bind to key conserved residues within the PLpro/ISG15 and PLpro/ubiquitin interfaces. Our findings demonstrate that nanobodies targeting the PLpro/ISG15 interface can achieve synergistic antiviral and immunomodulatory effects, providing a proof-of-concept for a novel therapeutic approach to combat SARS-CoV-2 and potentially other emerging coronaviruses.

**IMPORTANCE:**

The COVID-19 pandemic caused by SARS-CoV-2 has resulted in millions of deaths worldwide. Despite the emergence of antiviral drugs and vaccines targeting 3CLpro and RNA-dependent RNA polymerase (Rdrp), the virus’s continuous mutation underscores the need for novel therapeutic approaches that target highly conserved viral regions. PLpro is an attractive target due to its roles in viral replication and host immune regulation. However, research on nanobodies targeting PLpro remains in its infancy. This study provides significant insights into the antiviral and anti-inflammatory functions of two nanobodies, NbP1 and NbP2, which specifically disrupt the PLpro/ISG15 interaction interface. Our findings have important implications for drug development targeting SARS-CoV-2, highlighting the potential of nanobody-based therapeutics to simultaneously inhibit viral replication and suppress pathological inflammation.

## INTRODUCTION

Since the onset of the COVID-19 pandemic, nearly 800 million infections and millions of deaths have been reported worldwide. Furthermore, a significant proportion of convalescent patients suffer from post-acute sequelae of COVID-19 (PASC), imposing a substantial burden on global public health systems (1, 2). To date, FDA-approved antiviral therapies have primarily targeted the main protease (Mpro, also known as 3CLpro), the RNA-dependent RNA polymerase (RdRp), or the Spike protein receptor-binding domain (RBD) to prevent viral entry (3). However, the emergence of drug-resistant mutations in these essential enzymes is increasingly compromising the efficacy of current treatments (4, 5). Moreover, the rapid evolution of new variants has diminished the protective capacity of first-generation vaccines and neutralizing antibodies (6–8). Consequently, identifying highly conserved viral regions as targets for next-generation therapeutics has become a critical priority. In this context, the papain-like protease (PLpro), which is indispensable for the viral life cycle, has emerged as a highly promising therapeutic target.

SARS-CoV-2 utilizes two primary proteases, Mpro and PLpro, to cleave viral polyproteins (pp1a and pp1ab) into individual functional non-structural proteins (nsps) required for replication (9, 10). PLpro, a cysteine protease, specifically recognizes the conserved Leu-Xaa-Gly-Gly motif within the polyproteins, catalyzing the release of nsp1, nsp2, nsp3, and nsp4 (11). This proteolytic processing is essential for viral protein maturation, genome transcription, and assembly of the replicase-transcriptase complex. Beyond its role in replication, PLpro functions as a potent immune antagonist. It possesses deubiquitinating and de-ISGylating activities, allowing it to remove ubiquitin and interferon-stimulated gene 15 (ISG15) modifications from both host and viral substrates, such as the host sensor MDA5 and the viral nucleocapsid (N) protein (12, 13). By stripping these modifications, PLpro effectively suppresses the host’s type I interferon (IFN) signaling pathway, thereby facilitating immune evasion. Therefore, targeting PLpro offers a dual therapeutic advantage: inhibiting viral replication while restoring the host’s innate immune response and mitigating excessive inflammation.

Despite its therapeutic potential, the development of potent and selective PLpro inhibitors has encountered significant hurdles. The primary challenge lies in the structural homology between the catalytic domain of PLpro and several human deubiquitinating enzymes, particularly those of the USP (ubiquitin-specific protease) family. Achieving high selectivity is essential to avoid off-target toxicity in host cells (13–15). Most early efforts focused on small-molecule inhibitors derived from the GRL0617 scaffold, which binds to the S1/S2 pocket of PLpro (14, 16). While these molecules showed promise *in vitro*, many suffered from poor pharmacokinetic profiles or insufficient potency *in vivo*. Currently, progress in PLpro inhibitor development in clinical pipelines remains limited compared to Mpro inhibitors; for instance, HL-21 is one of the few candidates to have reached Phase II clinical trials (17, 18). Furthermore, the relatively shallow and solvent-exposed nature of the PLpro-binding pockets makes the design of high-affinity small molecules inherently difficult, often requiring complex optimization to achieve therapeutic concentrations without compromising safety (19).

Nanobodies, the single-domain variable regions derived from camelid heavy-chain antibodies, exhibit remarkable stability, unique binding modes, and the ability to effectively neutralize viral particles (20). Owing to their small size and modularity, nanobodies hold immense potential for diverse applications, including infectious disease diagnostics, intracellular delivery, and targeted therapy, establishing them as pivotal tools in modern biotechnology (21, 22). While numerous antibodies and nanobodies have been developed against SARS-CoV-2, the vast majority target the Spike protein (23–27). However, the high mutation rate of the Spike protein renders these biologics highly susceptible to viral escape. Thus, developing therapeutics against more conserved internal proteins like PLpro is essential for broad-spectrum efficacy.

In this study, we identified two nanobodies, NbP1 and NbP2, using yeast surface display screening, which exhibit potent inhibitory activity against PLpro. We characterized their antiviral and anti-inflammatory properties through biochemical assays, pseudovirus and authentic SARS-CoV-2 neutralization tests, and *in vitro* and *in vivo* models of intestinal inflammation. Notably, the TAT-Nb fusion also alleviated pulmonary inflammation in a K18-hACE2 mouse respiratory tract infection model induced by SARS-CoV-2 transcription-and-replication-competent virus-like particle (trVLPs). Structural epitope mapping revealed that these nanobodies bind to critical residues within the PLpro/ISG15 interaction interfaces. Our findings suggest that disrupting these interfaces is a key mechanism for achieving synergistic antiviral and immunomodulatory effects, providing a novel framework for the design of anti-SARS-CoV-2 therapeutics.

## RESULTS

### Screening and identification of PLpro-specific nanobodies via yeast surface display

**Fig 1:**
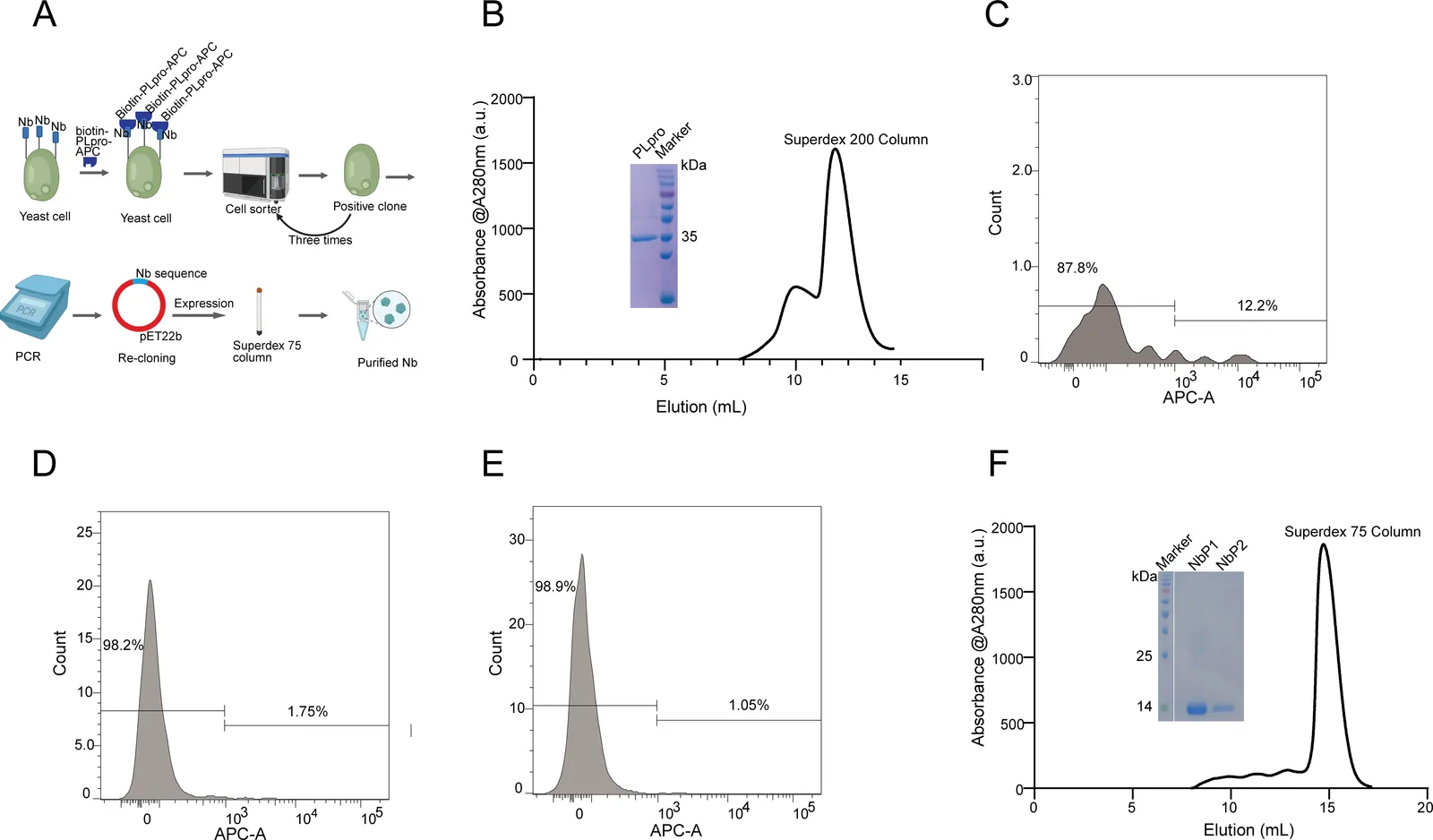
Identification of two nanobodies via yeast surface display screening. (**A**) Schematic representation of the yeast surface display screening workflow. (**B**) Size-exclusion chromatography profile and SDS-PAGE analysis of the purified PLpro protein. (**C–E**) Flow cytometric analysis shows the enrichment of the nanobody library from selection rounds 1 to 3. (**F**) Typical size-exclusion chromatography profile and SDS-PAGE analysis of the purified nanobodies NbP1 and NbP2.

To identify nanobodies targeting PLpro, we employed a yeast surface display system adapted from methods previously described by Kruse et al. (28). Briefly, a nanobody library was generated via over-extension PCR and cloned into the modified pPICZα vector. The library was then transformed into the *Pichia pastoris* strain X-33 through electroporation, and the screening process was conducted as illustrated in Fig. 1A. Purified PLpro protein (Fig. 1B) was biotinylated and incubated with the yeast library at decreasing concentrations (100 nM, 10 nM, and 1 nM) across successive rounds of selection. Following labeling with allophycocyanin (APC), yeast cells displaying PLpro-binding nanobodies were isolated using fluorescence-activated cell sorting (FACS), with the sorting profiles shown in Fig. 1C through E. Yeast populations obtained from each round were expanded and preserved for subsequent selection cycles. FACS analysis revealed enrichment rates of 12.2%, 1.75%, and 1.05% in the first, second, and third rounds, respectively. Following the third round of enrichment, the candidate sequences were subcloned into an *E. coli* expression vector for protein production. After expression and purification (Fig. 1F), two lead nanobodies, designated NbP1 and NbP2, were successfully identified and characterized.

### Nanobodies exhibit specific binding to PLpro independent of cell-penetrating peptide conjugation

**Fig 2:**
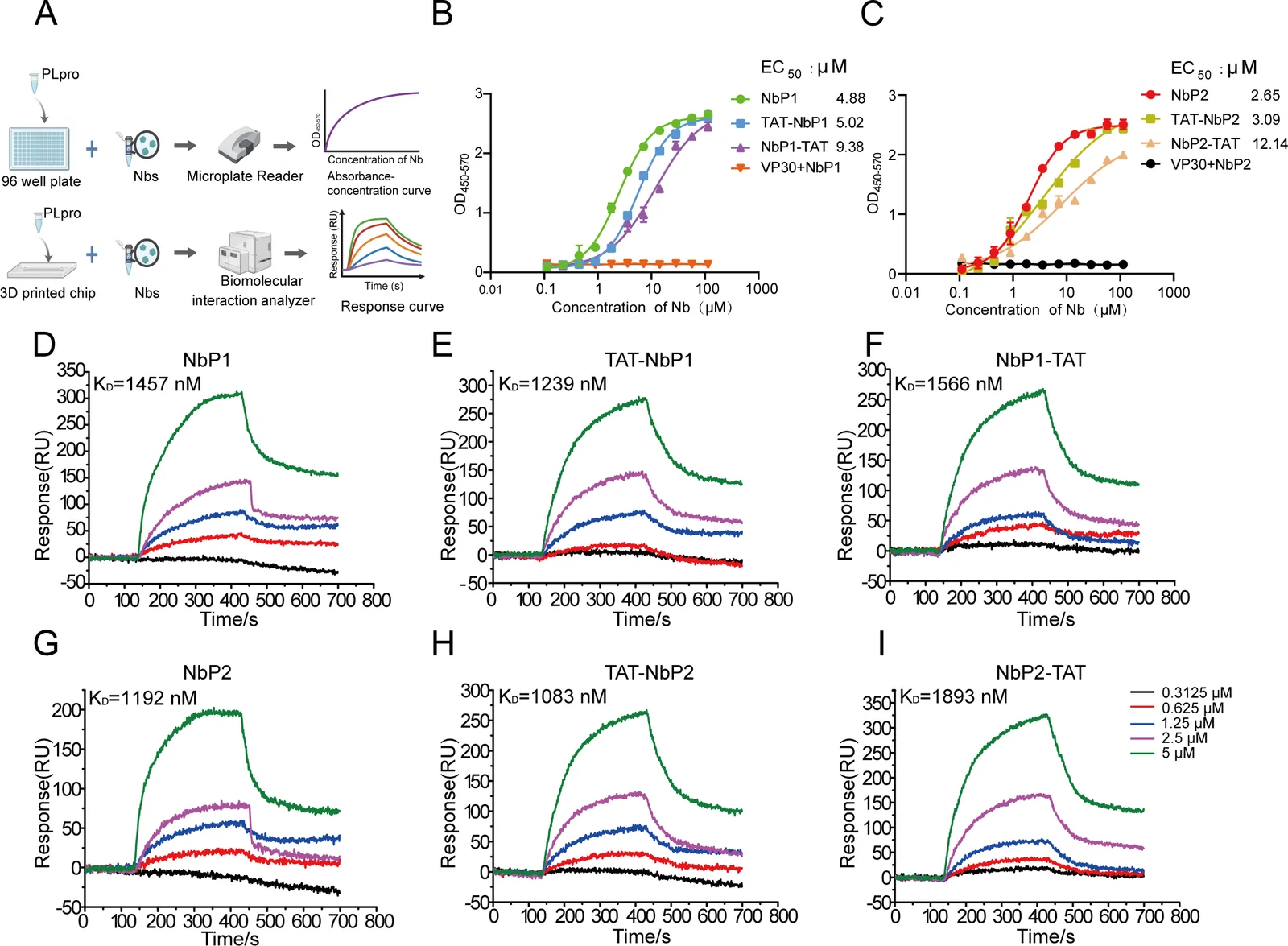
Binding affinity and activity of NbP1 and NbP2 fused with the TAT peptide. (**A**) Schematic illustration of the ELISA and SPR experimental workflows. (**B and C**) ELISA-based binding activity of NbP1 and NbP2 to PLpro, comparing nanobodies with and without TAT fusion. (**D–I**) SPR sensorgrams and affinity constants K_D_ of NbP1 and NbP2 interacting with PLpro, with and without the TAT cell-penetrating peptide.

To characterize the binding properties of the identified nanobodies, we performed enzyme-linked immunosorbent assay (ELISA) and surface plasmon resonance (SPR) assay (schematic shown in Fig. 2A). Initially, the binding affinities of NbP1 and NbP2 for PLpro were evaluated via ELISA, yielding EC_50_ values of 4.88 μM and 2.65 μM, respectively (Fig. 2B and C). To confirm specificity, we tested the nanobodies against the Ebola virus VP30 protein—a critical component of the viral transcription-replication complex—as a negative control. Both nanobodies exhibited negligible binding to VP30, confirming their specificity for PLpro (Fig. 2B).

Given that PLpro is an intracellular target and nanobodies typically possess limited membrane permeability, we conjugated the nanobodies with the TAT cell-penetrating peptide at either the N-terminus (TAT-NbP1/NbP2) or the C-terminus (NbP1/NbP2-TAT) (29). We then reassessed their binding affinities to determine whether the addition of the TAT sequence affected target recognition (Fig. 2B and C). The N-terminal fusion (TAT-NbP1/NbP2) maintained an affinity nearly identical to the parental nanobodies. In contrast, the C-terminal fusion (NbP1/NbP2-TAT) resulted in a slight decrease in affinity, suggesting that C-terminal modification may cause minor steric interference with the complementarity-determining region 3 (CDR3), which is often critical for nanobody-antigen interactions.

The binding kinetics were further validated using SPR (Fig. 2D through I). The SPR results were consistent with the ELISA data, demonstrating robust binding activity for both nanobodies. Furthermore, specificity was reinforced by SPR analysis using a sensor chip functionalized with Ebola VP30; even at concentrations as high as 50 μM, no significant binding to VP30 was observed (Fig. S1). Collectively, these results demonstrate that NbP1 and NbP2 specifically bind to PLpro with high affinity and that N-terminal TAT conjugation does not compromise their binding integrity.

### NbP1 and NbP2 inhibit the proteolytic activity of SARS-CoV-2 PLpro *in vitro*

To evaluate the inhibitory potential of the identified nanobodies against the enzymatic activity of SARS-CoV-2 PLpro, we utilized a fluorogenic peptide substrate, ALKGG-7-amino-4-methylcoumarin (ALKGG-AMC) (30, 31). This substrate mimics the C-terminal consensus sequence recognized by PLpro; upon cleavage of the peptide bond following the Gly-Gly motif, the AMC fluorophore is released from its quenched state, producing a quantifiable fluorescent signal (Fig. S2A). We hypothesized that nanobody binding to PLpro would sterically or conformationally hinder substrate access to the active site, thereby reducing the rate of AMC release.

Dose-response assays revealed that both NbP1 and NbP2, as well as their TAT-fused derivatives, effectively inhibited PLpro-mediated substrate cleavage. Specifically, NbP1 exhibited an IC₅₀ of 1.65 μM, while its N-terminal and C-terminal TAT-fusions (TAT-NbP1 and NbP1-TAT) yielded IC₅₀ values of 1.67 μM and 4.60 μM, respectively (Fig. S2B). Similarly, NbP2 demonstrated an IC₅₀ of 2.93 μM, with its TAT-fused counterparts, TAT-NbP2 and NbP2-TAT, showing IC₅₀ values of 2.86 μM and 6.18 μM, respectively (Fig. S2C).

Notably, the N-terminal fusion of the TAT peptide (TAT-Nb) appeared to preserve the inhibitory potency more effectively than the C-terminal fusion (Nb-TAT), which resulted in a slight reduction in activity. These biochemical data demonstrate that both nanobodies, in their parental and TAT-conjugated forms, maintain high inhibitory efficacy against the proteolytic function of PLpro *in vitro*. These findings provided the biochemical foundation for subsequent evaluations of their antiviral and anti-inflammatory activities in cellular and *in vivo* models.

### Nanobodies conjugated with cell-penetrating peptides exhibit potent antiviral activity against trVLP SARS-CoV-2

**Fig 3:**
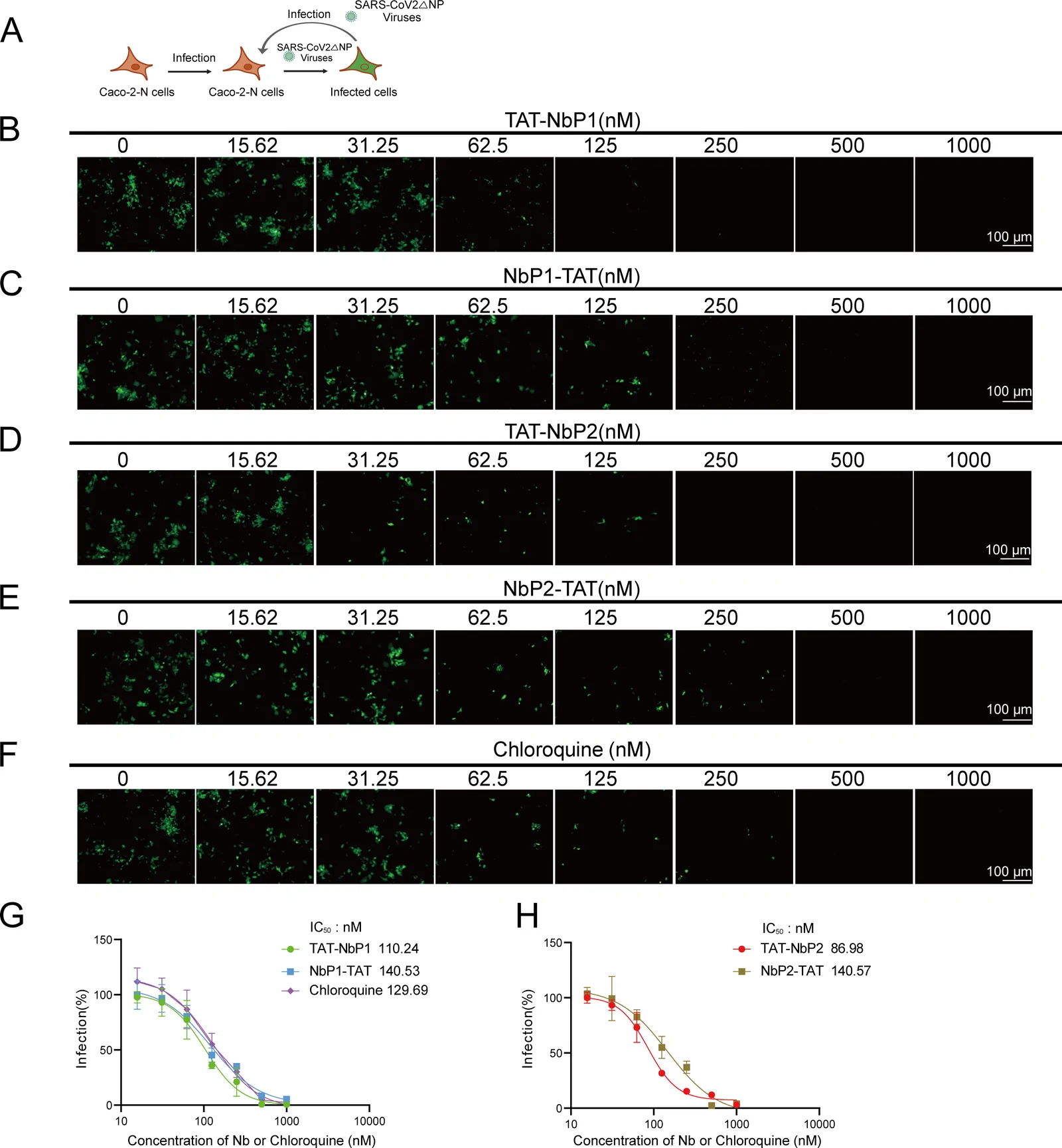
Inhibition of SARS-CoV-2 trVLP by TAT-fused NbP1 and NbP2. (**A**) Schematic representation of the SARS-CoV-2 trVLP infection and antiviral assay in Caco2-N cells. (**B–F**) Representative immunofluorescence images evaluating the antiviral activity of NbP1 and NbP2 (± TAT) and chloroquine against trVLP infection. Scale bar = 100 µm; *n* = 3 independent experiments. (**G–H**) Dose-response inhibition curves and calculated IC_50_ values for each inhibitor, determined by ImageJ-based quantification of fluorescence intensity.

To evaluate the antiviral potential of the identified nanobodies, we utilized a trVLP system (32), the mechanism of which is illustrated in Fig. 3A. Briefly, Caco2-N cells were infected with SARS-CoV-2ΔNP-EGFP. The viral inoculum was subsequently replaced with culture medium containing varying concentrations of nanobodies, and the cells were incubated for 48 h. Chloroquine, a known inhibitor of SARS-CoV-2 replication *in vitro*, served as a positive control (33), while a nanobody targeting the Ebola virus VP30 protein was used as a negative control.

Fluorescence microscopy (Fig. 3B through F) revealed that nanobodies fused with the TAT peptide (TAT-NbP1, NbP1-TAT, TAT-NbP2, and NbP2-TAT) significantly inhibited viral replication. Specifically, TAT-NbP1 and NbP1-TAT exhibited IC_50_ values of 110.24 nM and 140.53 nM, respectively (Fig. 3G). Similarly, TAT-NbP2 and NbP2-TAT showed potent antiviral activity with IC_50_ values of 86.98 nM and 140.57 nM, respectively (Fig. 3H). These results confirm the efficacy of TAT-mediated intracellular delivery. In contrast, the parental nanobodies lacking the TAT peptide exhibited negligible antiviral activity, likely due to their inability to traverse the host cell membrane. Furthermore, the TAT-fused VP30-targeting nanobody failed to reduce fluorescence, confirming that the observed antiviral effects were specific to PLpro inhibition (Fig. S3).

### TAT-conjugated nanobodies exhibit potent antiviral activity against authentic SARS-CoV-2 and EG.5 variants

**Fig 4:**
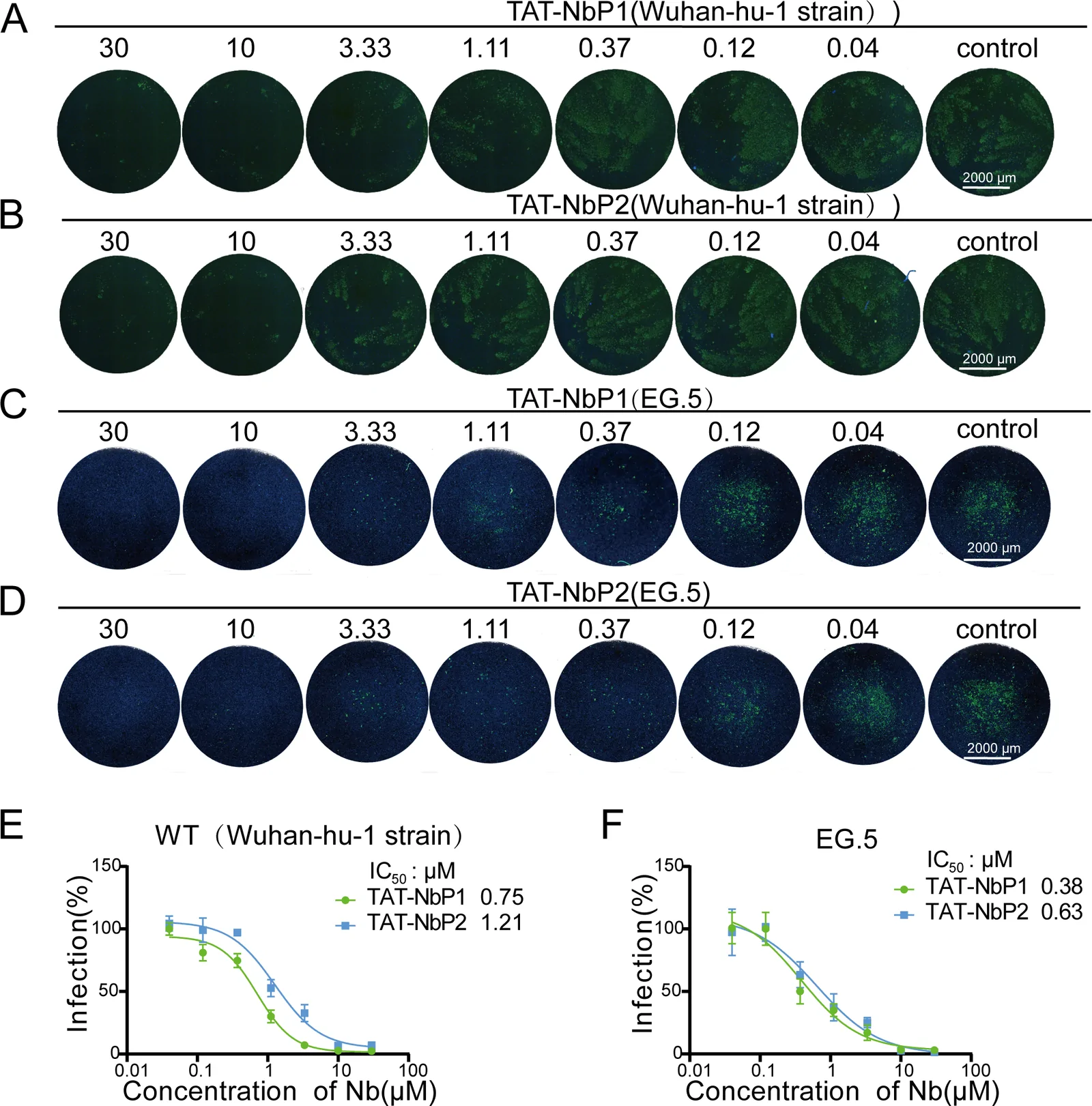
TAT-NbP1 and TAT-NbP2 effectively inhibit authentic SARS-CoV-2 strain Wuhan-Hu-1 and EG.5 variants. (**A–D**) Representative immunofluorescence images evaluating the antiviral activity of NbP1 and NbP2 (± TAT) and chloroquine against authentic SARS-CoV-2 (strain Wuhan-Hu-1) and the EG.5 variant. Scale bar = 2,000 µm; *n* = 3 independent experiments. (**E and F**) Dose-response inhibition curves and calculated IC_50_ values for each inhibitor, determined by ImageJ-based quantification of fluorescence intensity.

Following the validation of antiviral efficacy in the trVLP system, we evaluated the inhibitory capacity of the N-terminal TAT-tagged nanobodies (TAT-NbP1 and TAT-NbP2) against authentic SARS-CoV-2 strains, specifically the SARS-CoV-2 (strain Wuhan-Hu-1) and the EG.5 variant. Antiviral activity was assessed using immunofluorescence microscopy (Fig. 4A through D), with dose-response inhibition curves generated through fluorescence quantification (Fig. 4E and F). Similarly, immunofluorescence imaging revealed that the positive control chloroquine also maintained robust suppression of SARS-CoV-2 (strain Wuhan-Hu-1) and EG.5 variant replication (Fig. S4A and B), whereas TAT-CW056 (targeting Ebola VP30) did not exhibit any inhibitory effect on the replication of these viral strains, with chloroquine’s dose-response inhibition curves generated through fluorescence quantification (Fig. S4C and D).

Both nanobodies demonstrated robust inhibition of authentic viral replication. Against SARS-CoV-2 (strain Wuhan-Hu-1), TAT-NbP1 and TAT-NbP2 yielded IC_50_ values of 0.75 μM and 1.21 μM, respectively. Notably, both nanobodies maintained high potency against the EG.5 variant, exhibiting IC_50_ values of 0.38 μM and 0.63 μM, respectively. Similarly, the positive control chloroquine also potently inhibited the replication of the SARS-CoV-2 (strain Wuhan-Hu-1) and EG.5 strains, with IC₅₀ values of 0.39 and 0.47 μM, respectively. These findings indicate that NbP1 and NbP2 target highly conserved regions of PLpro, enabling them to retain efficacy against evolving SARS-CoV-2 variants.

### TAT-conjugated nanobodies disrupt the PLpro/ISG15 interface and attenuate PLpro-induced inflammation in NCM460 cells

**Fig 5:**
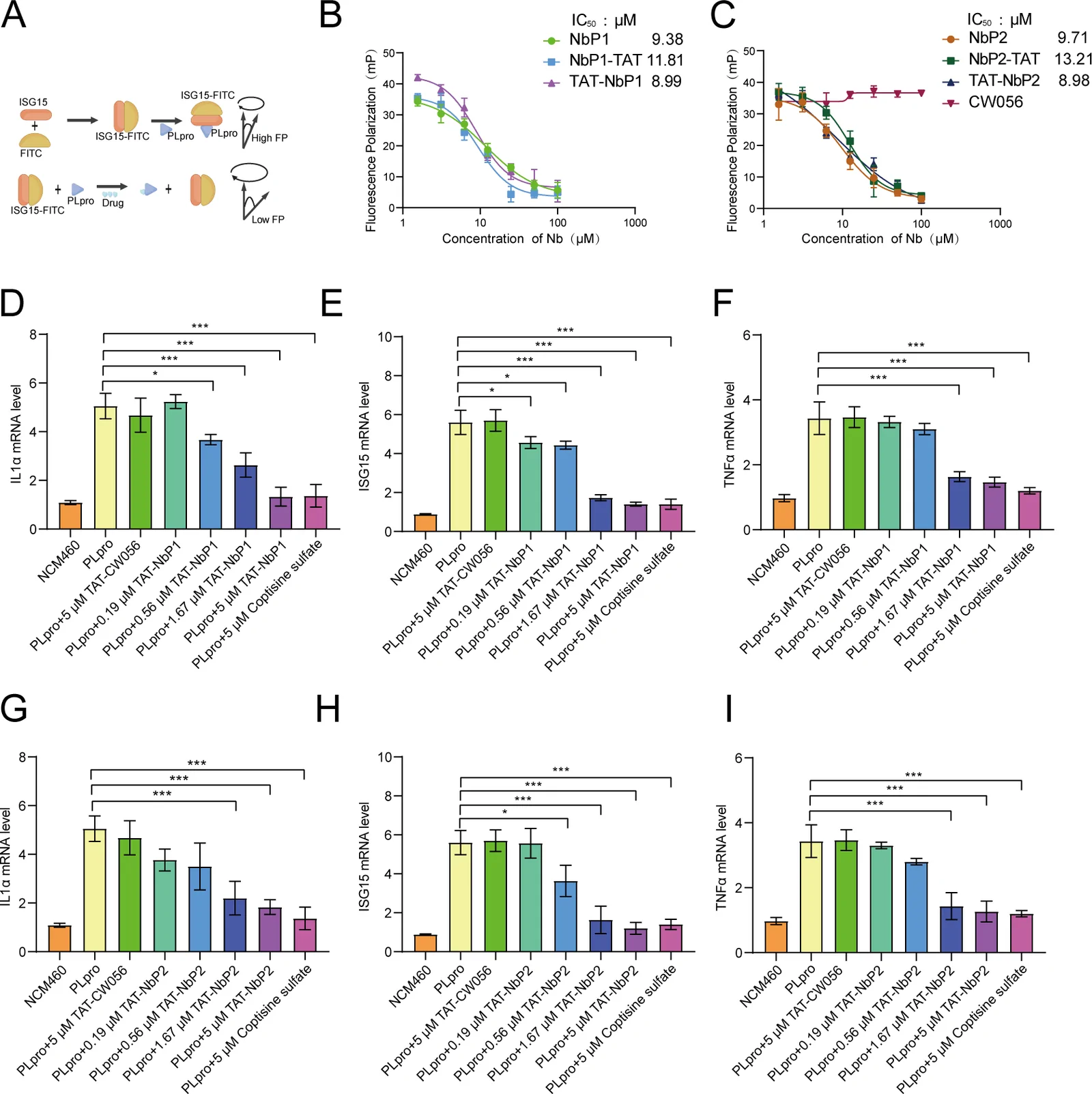
TAT-fused NbP1 and NbP2 attenuate PLpro-induced inflammatory responses in NCM460 cells by targeting the PLpro/ISG15 interface. (**A**) Schematic illustration of nanobody-mediated blockade of the PLpro/ISG15 binding interface. (**B and C**) Dose-response curves showing the *in vitro* inhibition of the PLpro/ISG15 interaction by NbP1 and NbP2 (± TAT). (**D–I**) Real-time quantitative PCR (RT-qPCR) analysis of *ISG15*, *IL-1α,* and *TNF-α* mRNA levels in NCM460 cells. TAT-fused NbP1 and NbP2 suppress the expression of these inflammatory mediators by blocking the PLpro/ISG15 interface. Data are expressed as mean ± SD (*n* = 3). Statistical significance was determined by one-way ANOVA: *P < 0.05, **P < 0.01, ***P < 0.001.

The interaction between PLpro and ISG15 facilitates the removal of ISG15 from host proteins, a process termed deisgylation that modulates the host inflammatory response (34). To determine whether the identified nanobodies could interfere with this process, competitive fluorescence polarization (FP) assays were employed to evaluate the ability of NbP1 and NbP2, in both parental and TAT-conjugated forms, to disrupt the PLpro/ISG15 binding interface. The principle of the competitive FP assay is illustrated in Fig. 5A, and the baseline polarization changes for the interaction between PLpro and fluorescein isothiocyanate (FITC)-labeled ISG15 (FITC-ISG15) are shown in Fig. S5A.

As shown in Fig. 5B, NbP1 effectively competed with ISG15 for the PLpro binding interface, yielding IC_50_ values of 9.38, 8.99, and 11.81 μM for the parental, N-terminal TAT, and C-terminal TAT versions, respectively. Similarly, NbP2 exhibited robust inhibitory activity with IC50 values of 9.71, 8.98, and 13.21 μM for the respective versions (Fig. 5C). In contrast, the negative control nanobody, CW056, which targets Ebola VP30, showed negligible interference with the PLpro/ISG15 interface (Fig. 5C). These results demonstrate that both nanobodies specifically target the ISG15-binding site on PLpro and that this binding capacity is maintained following TAT conjugation.

Previous studies have established that PLpro stimulation triggers the elevation of pro-inflammatory cytokines and ISG15 levels in NCM460 colonic epithelial cells and that targeting the PLpro/ISG15 interface can mitigate these effects (35). Consequently, the ability of N-terminal TAT-conjugated nanobodies to suppress PLpro-induced inflammation in NCM460 cells was investigated. RT-qPCR analysis revealed that both TAT-NbP1 and TAT-NbP2 significantly reduced the expression of TNF-alpha and IL-1alpha in a dose-dependent manner (Fig. 5D through I). Furthermore, a concurrent decrease in ISG15 mRNA levels was observed, suggesting that the anti-inflammatory effects of these nanobodies are directly linked to the disruption of the PLpro/ISG15 interaction. Coptisine sulfate, used as a positive control, effectively reversed the elevation of inflammatory markers, while the VP30-targeting nanobody had no effect. Collectively, these data indicate that TAT-NbP1 and TAT-NbP2 exert potent anti-inflammatory activity by inhibiting the de-ISGylating function of PLpro.

To further elucidate the mechanism by which these two nanobodies reverse elevated levels of inflammatory cytokines, SARS-CoV-2 trVLPs were utilized. Cells were stimulated with SARS-CoV-2 trVLP at a multiplicity of infection (MOI) of 0.5 for 12 h, followed by treatment with nanobodies at a final concentration of 5 μM for 36 h. Total RNA was subsequently extracted, and GFP levels were analyzed via RT-qPCR. The results demonstrated that while SARS-CoV-2 trVLP stimulation led to elevated GFP levels in the NCM460 cell line, treatment with the nanobodies resulted in a significant reduction in these levels (Fig. S5B). Given that trVLP is incapable of autonomous replication in NCM460 cells, the observed reduction in GFP suggests immune-mediated clearance. It is inferred that trVLP stimulation activates the innate immune response, which is subsequently suppressed by the de-ISGylating activity of PLpro within the trVLP. Treatment with the N-terminal TAT-fused nanobodies likely inhibits the de-ISGylating activity of PLpro, thereby restoring the innate immune pathway and promoting viral clearance, ultimately leading to the observed decrease in GFP levels.

### TAT-conjugated nanobodies attenuate colonic inflammation in a murine model

**Fig 6:**
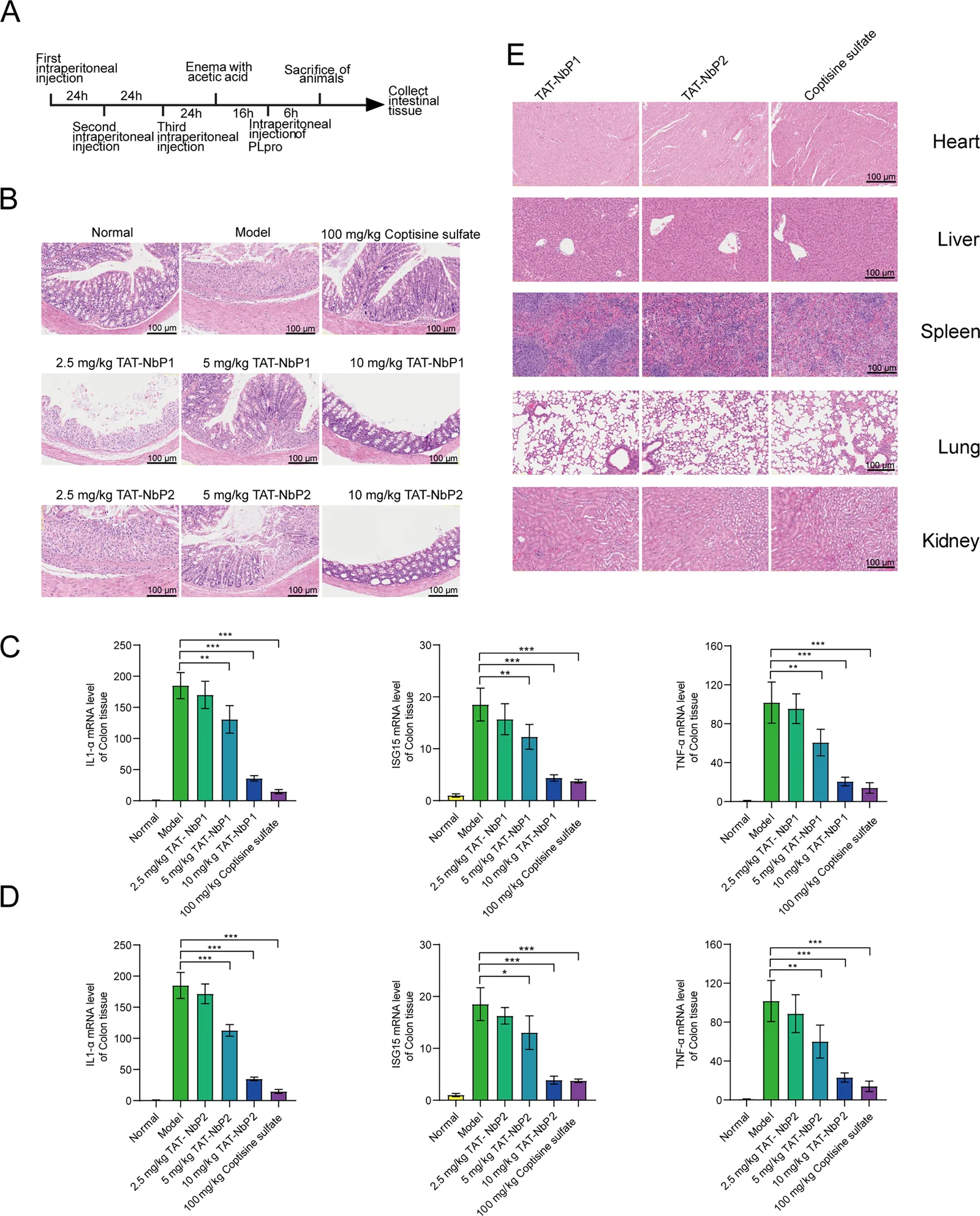
TAT-NbP1 and TAT-NbP2 ameliorate colitis induced by acetic acid and PLpro stimulation. (**A**) Schematic representation of the experimental design and treatment regimen in the mouse model of colitis. (**B**) Representative H&E-stained sections of colon tissues show the protective effects of TAT-NbP1 and TAT-NbP2 against colonic inflammation. Scale bar = 100 µm; *n* = 4. (**C and D**) RT-qPCR analysis of inflammatory cytokine expression in colon tissues following treatment with TAT-NbP1 and TAT-NbP2. (**E**) H&E staining of major organs (heart, liver, spleen, lung, and kidney) for systemic toxicity assessment of TAT-fused nanobodies. Scale bar = 100 µm; *n* = 4. Data are presented as mean ± SD. Statistical significance was determined by one-way ANOVA: *P < 0.05, **P < 0.01, ***P < 0.001.

To evaluate the therapeutic potential of the identified nanobodies *in vivo*, we administered N-terminal TAT-conjugated nanobodies to C57BL/6J female mice via intraperitoneal injection for three consecutive days at doses of 2.5, 5, and 10 mg/kg prior to the induction of colitis. The experimental timeline and dosing regimen are illustrated in Fig. 6A. Changes in mouse body weight throughout the animal experiment were recorded in Fig. S6. Following euthanasia, colonic tissues were harvested for histopathological evaluation using hematoxylin and eosin (H&E) staining.

As shown in Fig. 6B, the model group displayed significant pathological changes, including the loss of goblet cells and crypt architecture, accompanied by robust inflammatory cell infiltration. While the low- and medium-dose groups (2.5 and 5 mg/kg) showed moderate histopathological improvement, the high-dose group (10 mg/kg) and the positive control group exhibited substantial alleviation of these symptoms. These findings suggest that both nanobodies exert anti-inflammatory effects in a dose-dependent manner.

Consistent with the histopathological observations and *in vitro* data, RT-qPCR analysis (Fig. 6C and D) confirmed that nanobody treatment significantly reduced the expression of pro-inflammatory cytokines (TNF-α and IL-1α) at the site of inflammation. To further elucidate the underlying mechanism, we quantified *ISG15* expression, which followed a trend similar to that of the pro-inflammatory cytokines. PLpro-mediated de-ISGylation is known to suppress innate immunity and exacerbate inflammation; by disrupting the PLpro/ISG15 interface, TAT-NbP1 and TAT-NbP2 likely restore host innate immune signaling, thereby facilitating viral clearance and reducing the overall inflammatory burden. This is reflected in the decreased *ISG15* levels observed in the treated groups. Similarly, the positive control, coptisine sulfate, demonstrated a robust capacity to reverse colonic inflammation.

### N-terminal TAT-conjugated nanobodies attenuate SARS-CoV-2 trVLP-induced pulmonary inflammation in K18-hACE2 mice

**Fig 7:**
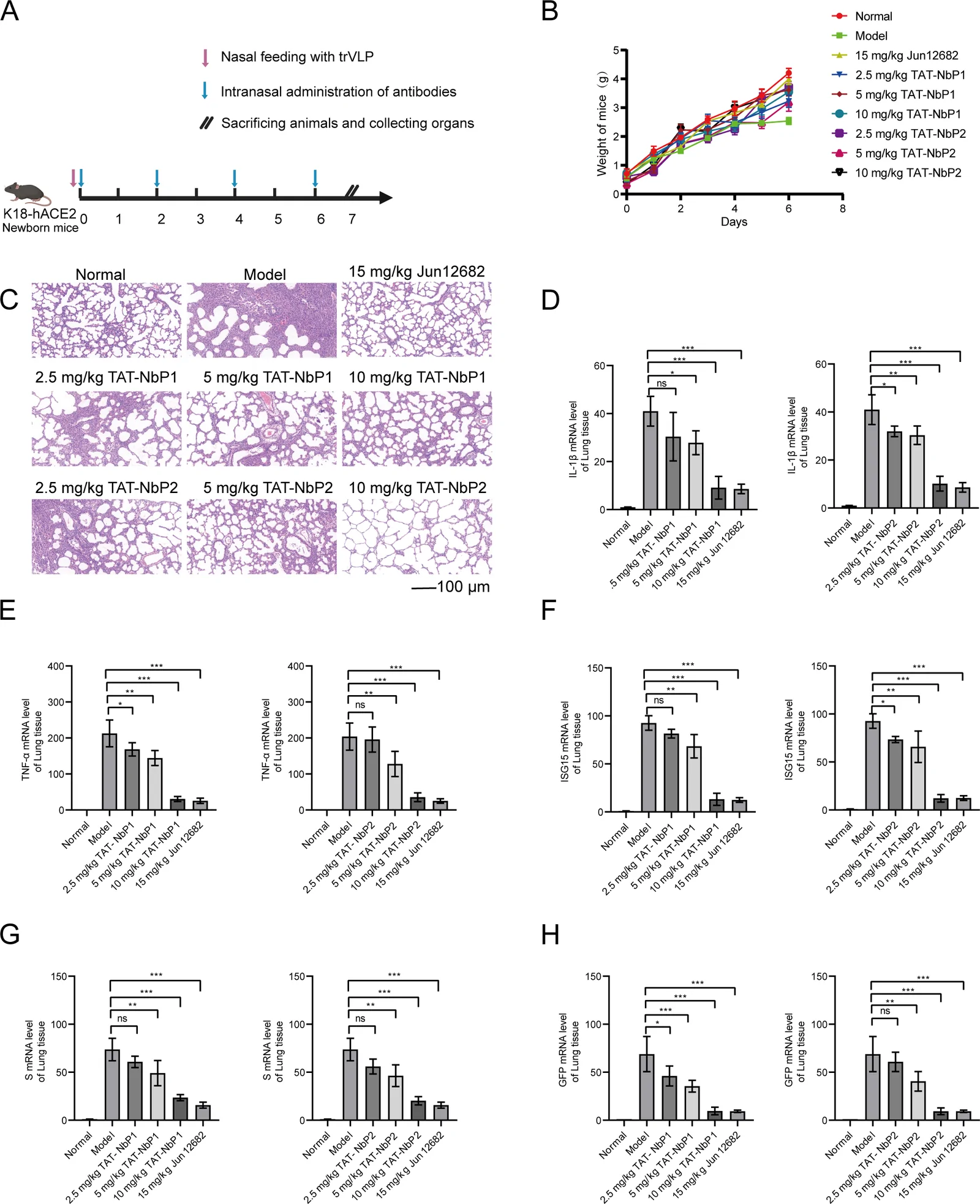
Therapeutic efficacy of TAT-fused nanobodies against SARS-CoV-2 trVLP in K18-hACE2 neonatal mice. (**A**) Experimental timeline of the SARS-CoV-2 trVLP challenge and nanobody treatment. (**B**) Body weight changes of K18-hACE2 neonatal mice recorded daily post-infection. (**C**) Representative images of H&E-stained lung tissue sections. (**D–H**) Relative mRNA expression levels of pro-inflammatory cytokines (**D–F**: IL-1β, TNF-α, and ISG15) and viral markers (**G and H**: S and GFP) in lung tissue determined by RT-qPCR. Data are presented as mean ± SD (*n* = 4). *P < 0.05, **P < 0.01, ***P < 0.001.

To evaluate the *in vivo* efficacy of the nanobodies within a SARS-CoV-2 respiratory infection framework, a SARS-CoV-2 trVLP challenge model was established in K18-hACE2 mice. This model was utilized to assess the protective potential of NbP1 and NbP2 conjugated to the N-terminal TAT peptide. The experimental procedure is delineated in Fig. 7A. Throughout the study period, the body weights of the mice were recorded daily, and no significant deviations in weight were observed across the experimental groups (Fig. 7B).

Histopathological analysis via H&E staining demonstrated that trVLP challenge induced substantial pulmonary damage, characterized by alveolar wall thickening and the recruitment of inflammatory cells. Notably, administration of the N-terminal TAT-fused nanobodies resulted in a dose-dependent mitigation of these pathological features. The high-dose treatment groups exhibited therapeutic efficacy comparable to the positive control, Jun12682, indicating that these nanobodies provide robust protection against trVLP-induced lung injury (Fig. 7C).

The expression profiles of inflammatory mediators in lung tissues were further quantified at the molecular level. RT-qPCR analysis revealed a marked elevation of TNF-α, IL-1β, and ISG15 mRNA levels in the trVLP-challenged model group. Conversely, treatment with the N-terminal TAT-fused nanobodies significantly downregulated the expression of these pro-inflammatory cytokines (Fig. 7D through F). Furthermore, mRNA levels of GFP and the Spike protein were measured as surrogate markers for viral presence (Fig. 7G and H). Consistent with the reduction in inflammatory markers, the levels of these viral transcripts were significantly diminished in the nanobody-treated groups compared to the model group.

These findings suggest that repeated trVLP exposure triggers innate immune activation and elevates ISG15 expression in the murine model. By inhibiting the de-ISGylating activity of PLpro within the trVLPs, the TAT-fused nanobodies likely suppress the cytokine storm while restoring innate immune signaling pathways to facilitate viral clearance. This dual mechanism effectively ameliorates pulmonary inflammation and reduces the levels of inflammatory cytokines and viral markers.

### TAT-conjugated nanobodies demonstrate minimal cytotoxicity *in vitro*

To evaluate the safety profile of the nanobodies, we performed 3-(4,5-dimethylthiazol-2-yl)−2,5-diphenyltetrazolium bromide (MTT) cytotoxicity assays on NCM460 cells using constructs both with and without the TAT peptide (Fig. S7A and B). The results indicated that the parental nanobodies (lacking the TAT peptide) exhibited negligible cytotoxicity, maintaining over 85% cell viability even at concentrations as high as 200 μM. Although conjugation of the TAT peptide slightly increased cytotoxicity, the effect remained minimal, with all half-maximal cytotoxic concentrations CC_50_ exceeding 100 μM. These findings confirm that TAT-mediated delivery is a safe and viable strategy for the intracellular transport of these nanobodies.

### Epitope mapping identifies key residues within the PLpro/ISG15 and PLpro/ubiquitin interfaces recognized by NbP1 and NbP2

**Fig 8:**
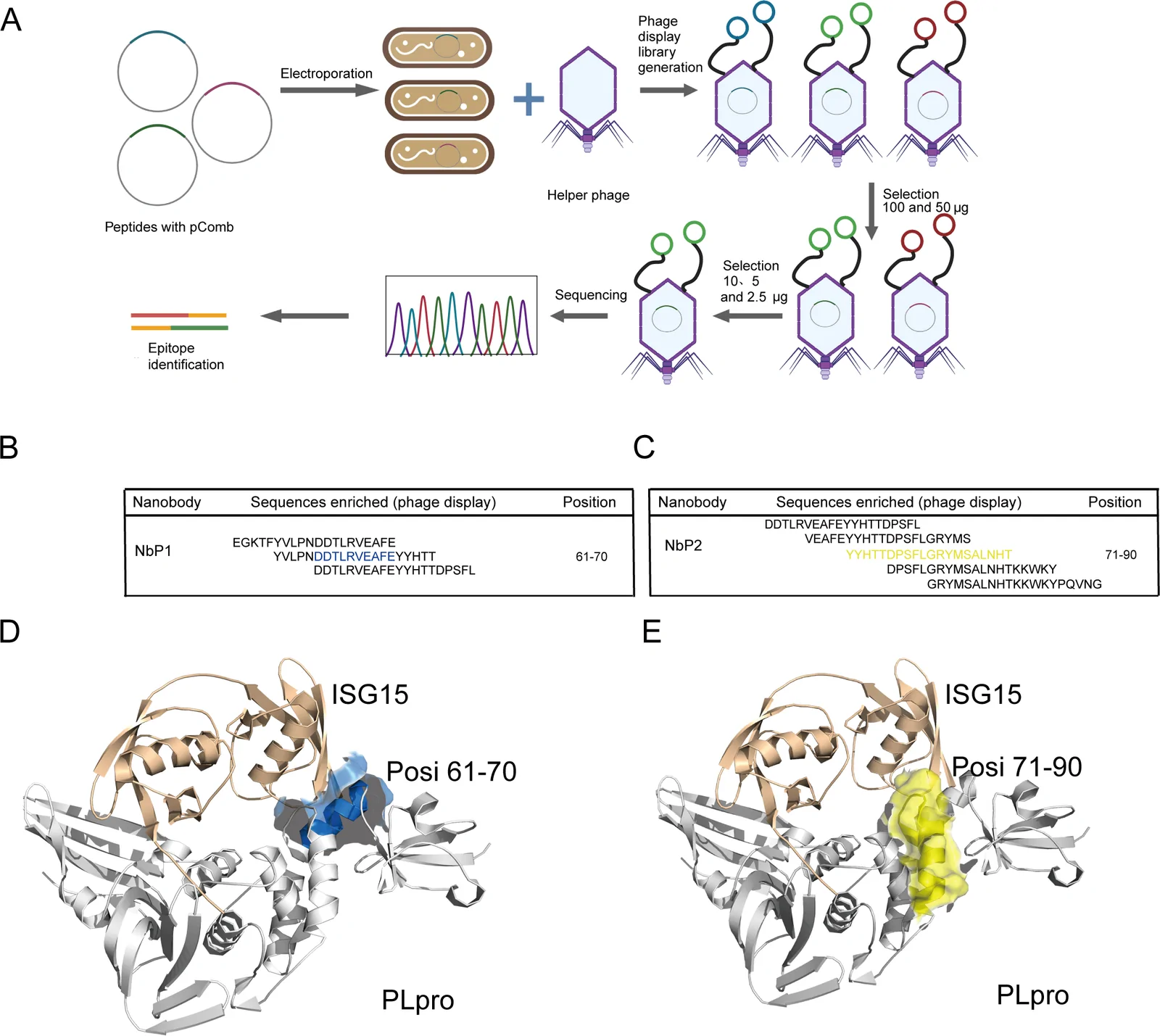
Structural mapping of NbP1- and NbP2-binding epitopes at the PLpro/ISG15 and PLpro/ubiquitin interfaces. (**A**) Schematic representation of epitope mapping using a phage display peptide library. (**B and D**) Identification of key residues involved in the NbP1–PLpro interaction and a structural representation of their molecular contacts. (**C and E**) Identification of key residues involved in the NbP2–PLpro interaction and a structural representation of their molecular contacts.

To define the specific binding interfaces of the nanobodies, we performed epitope mapping using a phage-display peptide library (Fig. 8A). This approach revealed that NbP1 and NbP2 recognize distinct epitopes on the PLpro protein. As illustrated in Fig. 8B through D, the peptide sequences enriched by NbP1 clustered within a primary region comprising residues 61–70.

Structural mapping onto the PLpro crystal structure indicated that these residues are located near the substrate-binding cleft. Specifically, Phe69 is a critical residue for the PLpro/ISG15 interaction but plays a negligible role in ubiquitin binding, while Glu70 was previously identified as a core residue involved in the interaction between PLpro and the distal ubiquitin moiety (14, 36). The spatial proximity of these residues in the folded protein suggests that NbP1 recognizes a conformational epitope spanning the Thumb and Palm domains.

In contrast, NbP2 targeted a distinct set of epitopes encompassing residues 71–90 (Fig. 8C through E). Within these regions, Thr75 is a key determinant of the low affinity of SARS-CoV-2 PLpro for ubiquitin chains (14).

In summary, high-throughput phage screening and sequence analysis demonstrate that NbP1 and NbP2 bind to discrete epitopes that encompass residues essential for the de-ISGylating and deubiquitinating activities of PLpro. These results provide a robust structural foundation for the potential development of bispecific antibodies or next-generation therapeutics designed to disrupt the PLpro/ISG15 and PLpro/ubiquitin interfaces.

## DISCUSSION

A significant proportion of COVID-19 patients present with gastrointestinal symptoms, the severity of which often correlates with increased morbidity. While the SARS-CoV-2 Spike protein has been implicated in triggering enteritis (37), recent evidence suggests that the PLpro also plays a critical role in intestinal pathogenesis. Specifically, PLpro-mediated cleavage releases free ISG15 into the extracellular space, where it acts as a cytokine-like molecule to stimulate pro-inflammatory signaling in T cells and NK cells (34). Our previous work established that PLpro induces colonic inflammation via the PLpro/ISG15 interaction interface and that disrupting this interface can alleviate inflammatory responses (35). While small-molecule inhibitors such as GRL0617 and YM155 have been developed to target the enzymatic activity of PLpro (38, 39), the use of nanobodies to target this protease remains largely unexplored.

In this study, we identified and characterized two nanobodies, NbP1 and NbP2, that provide a proof-of-concept for the dual-function targeting of PLpro. By utilizing yeast surface display, we isolated these nanobodies and validated their binding affinities in the low micromolar range. To overcome the challenge of intracellular delivery, we conjugated these nanobodies with the TAT cell-penetrating peptide. These TAT-fused constructs maintained robust binding to PLpro and demonstrated potent antiviral activity in both trVLP and authentic SARS-CoV-2 assays. Notably, TAT-NbP1 and TAT-NbP2 exhibited broad-spectrum efficacy, inhibiting both the SARS-CoV-2 Wuhan-Hu-1 strain and the EG.5 variant replication with IC_50_ values in the sub-micromolar to low-micromolar range.

The broad-spectrum potential of these nanobodies is supported by the high degree of structural conservation within the PLpro domain. Analysis of the PLpro sequence across major SARS-CoV-2 variants reveals that mutations are remarkably sparse within the catalytic core and the ISG15-binding interface. For instance, the Alpha variant contains no mutations in the PLpro domain, while the active sites of the Beta, Gamma, and Delta variants remain entirely conserved. Although the Omicron variant carries the K232Q mutation and the BA.2.76 sublineage includes a T24I mutation, these alterations reside outside the primary epitopes recognized by NbP1 and NbP2 (31, 40, 41). Consequently, these nanobodies are theoretically capable of neutralizing all current SARS-CoV-2 variants of concern. Furthermore, the 83% sequence similarity between the PLpro of SARS-CoV-2 and SARS-CoV suggests that these nanobodies may possess cross-reactive inhibitory potential against the original SARS-CoV, albeit potentially with reduced potency. This conservation is a stark contrast to the Spike protein, which frequently undergoes mutational escape. The success of broad-spectrum small molecules like F0213 against diverse coronaviruses, including MERS-CoV and seasonal hCoVs, underscores the viability of PLpro as a pan-coronavirus target (42–44). Nanobodies offer a distinct advantage in this context, as their small size and high specificity allow them to access conserved cryptic clefts that are often inaccessible to conventional antibodies or larger inhibitors.

A key finding of this study is the ability of these nanobodies to exert synergistic antiviral and anti-inflammatory effects. This dual mechanism was validated using a murine model of PLpro-induced inflammation, which provides unique insights compared to traditional acute infection models. While acute models typically focus on viral load and survival rates during the initial stages of infection (45, 46), our model specifically addresses the persistent inflammatory signaling that contributes to long-term sequelae, often referred to as Long-COVID (35). Recent progress in Long-COVID research suggests that the continued presence of viral proteins, such as PLpro, can drive chronic immune dysregulation even after active viral replication has subsided. By using a model that emphasizes cytokine storms and tissue damage rather than immediate lethality, we demonstrated that disrupting the PLpro/ISG15 interface directly mitigates the chronic inflammatory state. This approach is particularly relevant given that many COVID-19 survivors suffer from prolonged respiratory and gastrointestinal distress. Our results demonstrate that TAT-NbP1 and TAT-NbP2 effectively reversed pulmonary inflammation, exhibiting an efficacy comparable to that of the small-molecule inhibitor Jun12682. These findings underscore the therapeutic potential of targeting host-pathogen protein-protein interactions (PPIs) for the treatment of both the acute and post-acute phases of the disease.

The observation that TAT-NbP1 and TAT-NbP2 exhibit binding affinities in the micromolar range warrants further consideration. While therapeutic antibodies typically target nanomolar or picomolar affinities, our results demonstrate that micromolar binding is sufficient to achieve potent biological inhibition *in vivo*. Several factors may contribute to this efficacy. First, the TAT peptide facilitates intracellular delivery and likely increases the local concentration of the nanobodies, effectively driving the equilibrium toward the bound state despite a higher dissociation constant. Second, the inhibitory potential of a PPI antagonist often depends more on the precise targeting of critical residues than on absolute affinity. By blocking hotspots such as Phe69, Glu70, and Thr75, NbP1 and NbP2 disrupt the essential architecture of the PLpro/ISG15 and PLpro/ubiquitin complexes, thereby halting de-ISGylation and deubiquitination. Furthermore, the inherent stability of nanobodies within the intracellular environment may allow for sustained inhibitory activity. While affinity maturation could further enhance their potency, these results provide a robust proof-of-concept that moderate-affinity nanobodies can exert significant therapeutic effects by precisely targeting functional protein interfaces.

Epitope mapping further elucidated the structural basis of this activity, confirming that NbP1 and NbP2 bind to critical residues within the conserved PLpro/ISG15 and PLpro/ubiquitin interaction interfaces (14, 36). Safety remains a paramount concern for intracellular biologics; importantly, TAT-NbP1 and TAT-NbP2 exhibited minimal cytotoxicity in colonic epithelial cells and no detectable systemic toxicity in major organs at therapeutic doses.

Despite these promising results, several limitations remain to be addressed. While micromolar affinities were sufficient for our current biochemical and cellular models, clinical-grade therapeutics typically require nanomolar potency to ensure high efficacy at lower doses. Future research should prioritize high-resolution structural determination via X-ray crystallography or cryo-electron microscopy to define the exact atomic interactions within these epitopes. Such insights will enable structure-guided design and directed evolution to optimize binding kinetics. Additionally, the pharmacokinetic profile of these candidates requires refinement. Due to their small size (~15 kDa), nanobodies are susceptible to rapid renal clearance, resulting in a short systemic half-life. Fusing these candidates to human IgG Fc fragments could increase their molecular weight above the glomerular filtration threshold and leverage FcRn-mediated recycling to extend serum persistence while potentially adding immune effector functions (47). Finally, although the targeted epitopes appear highly conserved, our experimental validation was primarily limited to SARS-CoV-2 and its variants. While structural homology suggests broad-spectrum potential across the Sarbecovirus subgenus, empirical testing against other highly pathogenic strains, such as SARS-CoV and MERS-CoV, is necessary to confirm pan-coronavirus activity. Addressing these challenges will be critical for transitioning these nanobodies into a robust therapeutic platform capable of addressing both current and emerging coronavirus threats. A critical limitation of the current study pertains to the *in vivo* animal model employed to evaluate the protective efficacy of TAT-NbP1 and TAT-NbP2 against SARS-CoV-2. We utilized a trVLP challenge system in K18-hACE2 neonatal mice, in which the viral N gene of SARS-CoV-2 was replaced by an EGFP reporter (SARS-CoV-2ΔN-EGFP) (32). This trVLP system was developed to enable SARS-CoV-2 research under BSL-2 conditions while retaining the complete set of viral structural proteins (S, E, and M) alongside a fluorescent reporter (32). The trVLP particles are capable of entry, transcription, and single-round expression of the reporter gene; however, they cannot complete a full replication cycle and produce progeny virions unless the N protein is supplied in *trans*. In our *in vitro* studies, this requirement was satisfied by using Caco2-N cells, which stably express the SARS-CoV-2 N protein and thereby permit trVLP to undergo complete replication cycles (32). In contrast, the K18-hACE2 mice used in our *in vivo* challenge model express the human ACE2 receptor but do not provide viral N protein. Consequently, the trVLP in our *in vivo* model can infect lung epithelial cells and deliver PLpro to trigger inflammatory responses, but cannot undergo multi-cycle viral replication or cell-to-cell spread. This fundamentally distinguishes our *in vivo* model from authentic SARS-CoV-2 infection, in which the complete viral life cycle, including genome replication, N protein-dependent virion assembly, budding, and progressive spread through the respiratory tract, contributes to disease pathogenesis. It is important to emphasize that this limitation does not invalidate our anti-inflammatory findings. Because the trVLP carries the complete set of SARS-CoV-2 structural proteins (S, E, and M) and PLpro, it retains the capacity to engage host immune pathways and induce inflammatory cascades that closely mimic aspects of authentic viral infection. Indeed, our data demonstrated that TAT-NbP1 and TAT-NbP2 effectively attenuated trVLP-induced pulmonary inflammation, as evidenced by reduced expression of TNF-α, IL-1β, and ISG15, along with improved histopathology. These findings are therefore highly relevant for evaluating the anti-inflammatory and immunomodulatory properties of our nanobodies. However, for assessing direct antiviral efficacy against viral replication and transmission, which are central to the therapeutic potential of PLpro-targeting nanobodies, the single-round nature of the trVLP in the absence of N protein complementation represents an incomplete model. Authentic SARS-CoV-2 *in vivo* challenge studies using live virus in appropriate animal models (e.g., K18-hACE2 mice or hamsters) are required to evaluate whether our nanobodies can reduce viral loads, limit pulmonary viral spread, and improve survival outcomes during multi-cycle infection. The use of the trVLP system for our *in vivo* studies was motivated by practical and biosafety considerations. Authentic SARS-CoV-2 *in vivo* challenge studies require Biosafety Level 3 (BSL-3) animal facilities, which are scarce, expensive to maintain, and involve extensive regulatory oversight. The trVLP system, as originally described by Ju et al. (32), was specifically developed to circumvent these constraints by enabling SARS-CoV-2-related research under BSL-2 conditions. This approach has been widely adopted for antiviral screening and mechanistic studies (32), and analogous N-complementation strategies have been applied to other enveloped viruses to enable biosafe replication-competent systems. Nevertheless, we acknowledge that authentic SARS-CoV-2 *in vivo* challenge experiments remain the gold standard for evaluating antiviral therapeutics. We plan to prioritize such studies in BSL-3 animal models as the next critical step to validate the *in vivo* antiviral efficacy of TAT-NbP1 and TAT-NbP2 under conditions that fully recapitulate SARS-CoV-2 pathogenesis.

**Fig 9:**
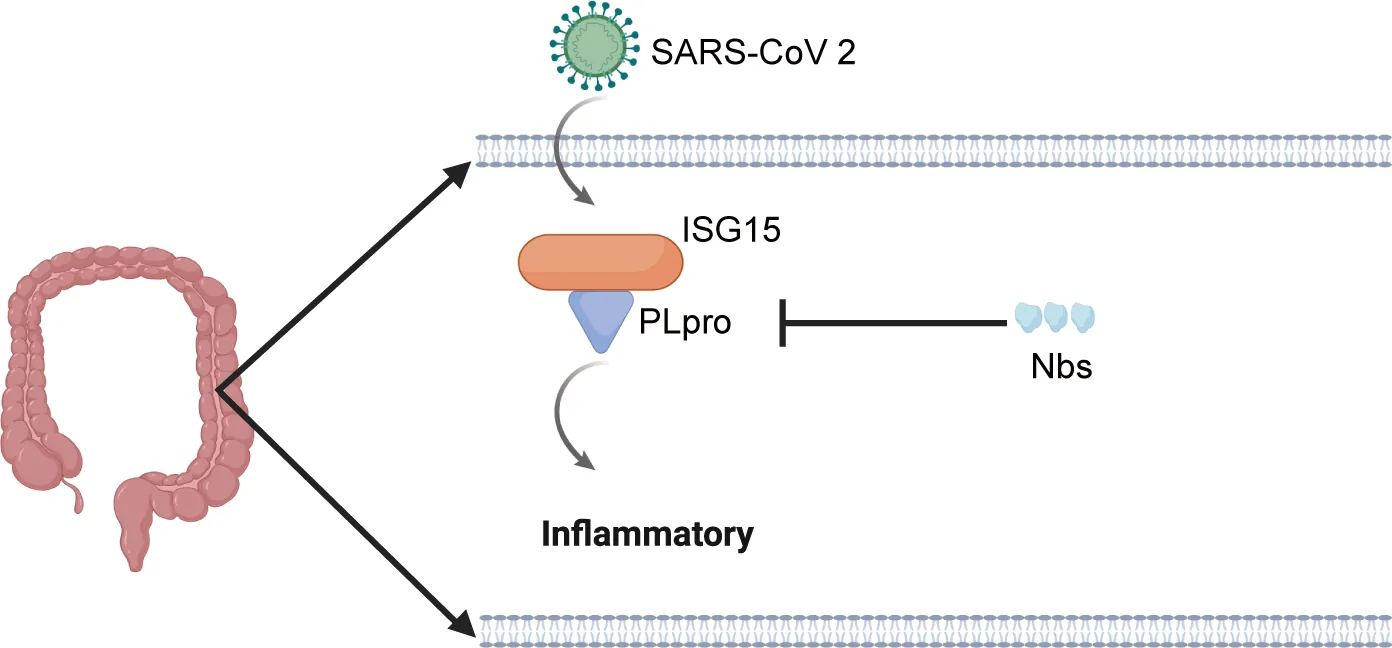
Graphical summary of nanobody-mediated inhibition of PLpro-induced enteric inflammation. The interaction between SARS-CoV-2 PLpro and ISG15 drives the progression of intestinal inflammation. Nanobodies designed to target this binding interface provide a therapeutic strategy to block the PLpro/ISG15 interaction and alleviate inflammatory symptoms.

In conclusion, this study identified and characterized two novel nanobodies, NbP1 and NbP2, targeting the SARS-CoV-2 PLpro. By utilizing TAT-mediated intracellular delivery, these nanobodies demonstrated potent antiviral activity against both the Wuhan-Hu-1 and EG.5 variants. Beyond direct viral inhibition, our findings reveal that these nanobodies effectively disrupt the PLpro/ISG15 and PLpro/ubiquitin interaction interfaces, thereby attenuating inflammatory responses in both cellular and murine models (Fig. 9). This work establishes a framework for the development of bifunctional therapeutics that simultaneously suppress viral replication and modulate host immunity, providing a promising strategy against current and future coronavirus threats.

## MATERIALS AND METHODS

### PLpro protein expression and purification

The gene encoding SARS-CoV-2 PLpro was subcloned into the pET28a vector and expressed in *Escherichia coli* BL21(DE3). Protein expression was induced with 0.5 mM isopropyl β-D-1-thiogalactopyranoside (IPTG; Beyotime) at 18°C for 16 h. Cells were harvested by centrifugation at 6,000 × *g* for 15 min at 4°C, resuspended in phosphate-buffered saline (PBS), and lysed using an ultrasonic cell disruptor (Scientz) at 33% amplitude.

The resulting lysate was clarified by centrifugation at 25,000 × *g* for 15 min at 4°C. The supernatant was then subjected to a multi-step purification process. First, hydrophobic interaction chromatography using a Phenyl column, followed by anion exchange chromatography using a Q Sepharose HP column to remove host cell protein contaminants. Final polishing was performed via size-exclusion chromatography using a Superdex 200 Increase column (Cytiva, USA) equilibrated in PBS. Protein purity and molecular weight were monitored throughout the purification process using sodium dodecyl sulfate-polyacrylamide gel electrophoresis (SDS-PAGE).

### Nanobody screening, expression, and purification

Nanobodies targeting PLpro were identified using a yeast surface display platform adapted from Kruse et al. A synthetic nanobody library was generated via overlap extension PCR (OE-PCR) using primers P1–P10 (Suzhou Hongxun Biotechnologies). Amplicons were cloned into a modified pPICZα vector, featuring an N-terminal α-mating factor secretion signal and a C-terminal GPI anchor for surface display. The library was then transformed into *Pichia pastoris* X-33 via electroporation.

For the selection process, purified PLpro was biotinylated using NHS-PEG12-Biotin (Thermo Fisher Scientific). Nanobody expression was induced on the yeast surface with methanol. The yeast library was incubated with varying concentrations of biotinylated PLpro, followed by labeling with APC-conjugated streptavidin. Antigen-binding clones were isolated via FACS using a FACS Aria flow cytometer (Becton Dickinson). The sorted populations were expanded and subjected to three successive rounds of enrichment.

Following the final round of selection, yeast clones were lysed via heat treatment (95°C for 10 min), and the nanobody sequences were amplified by colony PCR. The products were subcloned into the pET22b expression vector. After excluding sequences containing premature stop codons, the confirmed plasmids were transformed into *E. coli* BL21(DE3)-competent cells (Tsingke). Protein expression was induced with 0.5 mM IPTG at 16°C for 14 h. Cells were harvested, lysed, and the clarified supernatant was purified via immobilized metal affinity chromatography (IMAC) using a Ni-NTA column, followed by elution with an imidazole gradient. Final purification was achieved through size-exclusion chromatography using a Superdex 75 Increase 10/300 GL column (Cytiva, USA).

For TAT-conjugated nanobodies, the purification protocol was identical to that of the parental nanobodies. Protein purity was monitored throughout the process using SDS-PAGE. The primers utilized for TAT fusion are detailed in Table S1.

### Enzyme-linked immunosorbent assay

The binding affinity of the selected nanobodies for PLpro was determined via indirect ELISA. Briefly, 96-well half-area plates (Corning) were coated with 5 μg/well of purified PLpro antigen overnight at 4°C. The wells were then blocked with 5% non-fat dry milk in PBS for 1 h at room temperature. Subsequently, the plates were incubated with twofold serial dilutions of the nanobodies (ranging from 0.11 to 114 μM) for 1 h. After washing, an anti-6×His tag primary antibody (1:5,000) and a horseradish peroxidase (HRP)-conjugated mouse secondary antibody (1:3,000; Abclonal) were added sequentially. The colorimetric reaction was developed using TMB substrate (Beyotime) and monitored at 450 nm and 570 nm using a Tecan Spark microplate reader. Data are expressed as the difference in optical density (OD_450–570_).

### Surface plasmon resonance

The binding kinetics between PLpro and the nanobodies were analyzed using the PlexArray HT high-throughput surface plasmon resonance imaging (SPRi) system (Plexera, USA). Purified PLpro was covalently immobilized as the ligand on a 3D dextran sensor chip. Nanobodies, serving as the analytes, were injected over the chip surface at a series of concentrations. The association and dissociation phases were monitored in real time. The resulting sensograms were processed and fitted using Plexera Data Explorer software to determine the kinetic parameters, including the association rate constant (k_a_), dissociation rate constant (k_d_), and the equilibrium dissociation constant (K_D_).

### PLpro proteolytic inhibition assay

The inhibitory effect of nanobodies on PLpro activity was evaluated using a fluorogenic peptide substrate, ALKGG-AMC, representing the SARS-CoV-2 nsp3–nsp4 cleavage site. The peptide (Gier Biochemical, Shanghai; >98% purity) was prepared in Buffer A (50 mM HEPES, pH 7.5, 5 mM DTT, and 0.1 mg/mL BSA) at a 10 mM stock concentration. In the assay, 50 μL of 100 nM PLpro was incubated with 50 μL of serially diluted nanobodies (0.39–50 μM) for 10 min at room temperature. The reaction was initiated by adding 50 μL of 125 μM ALKGG-AMC. After 10 min, the reaction was quenched with 50 μL of 0.2 M sodium acetate. End-point fluorescence was measured at 25°C using a Tecan microplate reader (Ex/Em: 340/460 nm). Two controls were included: a positive control (buffer instead of nanobodies) and a negative control (no PLpro). All experiments were performed in triplicate.

### SARS-CoV-2 trVLP antiviral assay

Antiviral activity was evaluated using a transcription- and replication-competent SARS-CoV-2 virus-like particle system, kindly provided by Prof. Qiang Ding (Tsinghua University). This system utilizes a SARS-CoV-2 genome where the N gene is replaced by an enhanced green fluorescent protein (EGFP) reporter (SARS-CoV-2ΔN-EGFP), restricting viral replication to Caco2-N cells that provide the N protein in *trans* (32).

Caco2-N cells were maintained in high-glucose DMEM (Vigonob) supplemented with 5% fetal bovine serum (FBS). For the inhibition assay, cells were infected with trVLPs for 1 h at 37°C. The inoculum was then replaced with fresh medium containing serial dilutions of nanobodies and incubated for 48 h. EGFP expression was visualized via fluorescence microscopy, and total fluorescence intensity was quantified using ImageJ (NIH, USA). IC_50_ values were determined by fitting dose-response data to a non-linear regression model using GraphPad Prism 6 (GraphPad Software, USA).

### Authentic SARS-CoV-2 inhibition assays

All experiments involving authentic SARS-CoV-2 were conducted in a BSL-3 facility. To evaluate the antiviral efficacy of the nanobodies, Vero E6 cells were seeded in 96-well plates at a density of 2 × 10⁴ cells/well and cultured for 24 h. Cells were then inoculated with 50 μL of SARS-CoV-2 (Wuhan-Hu-1 strain or EG.5 variant) at an MOI of 0.05 and incubated at 37°C for 1 h. After removal of the viral inoculum, cells were treated with 100 μL of serially diluted nanobodies and incubated for an additional 24 h at 37°C.

Following incubation, cells were fixed with 4% paraformaldehyde and permeabilized with 0.2% Triton X-100. The viral N protein was detected via immunofluorescence using a rabbit anti-SARS-CoV-2 N protein polyclonal antibody (Sino Biological, Cat: 40143-T62), followed by an Alexa Fluor 488-conjugated anti-rabbit secondary antibody (Jackson). Nuclei were counterstained with DAPI (Sigma). Fluorescence signals were quantified using a Nexcelom Celigo automated imaging cytometer to determine the percentage of infected cells. IC_50_ values were calculated based on the reduction in fluorescence intensity relative to the virus-only control group.

### Fluorescence polarization analysis

FP assays were performed in black 96-well plates (Beyotime) to characterize the interaction between PLpro and ISG15. To determine the binding affinity, serial dilutions of PLpro (0.02–5 μM) were mixed with FITC-labeled ISG15 (final concentration: 100 nM) in a reaction buffer containing 25 mM NaH₂PO₄, 150 mM NaCl, 0.1 mg/mL BSA, and 7.5 μM ZnCl_2_. The ISG15 labeling procedure was conducted as previously described (30). Following a 30-min incubation at room temperature in the dark, FP signals were recorded using a Tecan Spark multimode microplate reader (λ_ex_ = 485 nm, λ_em_ = 535 nm).

For competitive FP assays, nanobodies (with or without TAT conjugation) were pre-incubated with PLpro (final concentration: 1 μM) for 30 min at room temperature. Subsequently, ISG15-FITC was added to a final concentration of 100 nM, and the mixture was incubated for an additional 1 h in the dark prior to measurement. Negative controls included an irrelevant nanobody targeting Ebola VP30 (isotype control) and the PLpro storage buffer (protein-free control). The IC_50_ values for the competitive inhibition of the PLpro/ISG15 interaction were calculated using non-linear regression analysis in GraphPad Prism 6.

### Evaluation of anti-inflammatory activity in NCM460 cells

NCM460 cells were maintained in RPMI 1640 medium (Gibco, USA) supplemented with 10% FBS. Cells were seeded in 6-well plates at a density of 6 × 10⁵ cells/well and incubated overnight at 37°C under 5% CO₂. To induce an inflammatory response, cells were stimulated with 5 μM purified PLpro in DMEM (10% FBS) for 36 h. Subsequently, the anti-inflammatory potential of the nanobodies was evaluated by adding TAT-conjugated constructs in a threefold serial dilution (0.18–5.0 μM) for an additional 36 h. Coptisine sulfate and TAT-CW056 (an anti-Ebola VP30 nanobody) served as positive and negative controls, respectively.

In parallel experiments to assess the impact on viral-induced inflammation, NCM460 cells were infected with SARS-CoV-2 trVLPs at an MOI of 0.5. After 12 h of infection, cells were treated with 5 μM nanobodies for 36 h. Total RNA was extracted using the Cell Total RNA Isolation Kit (Foregene, China), and RT-qPCR was performed on a LightCycler 480 II system (Roche, Switzerland). Relative mRNA expression levels of pro-inflammatory cytokines (*TNF-α* and *IL-1α*), ISG15, and *GFP* (for trVLP quantification) were calculated using the 2^−ΔΔCt^ method.

### *In vitro* cytotoxicity assay

The cytotoxicity of the nanobodies was evaluated in NCM460 cells using the MTT assay. Cells were seeded in 96-well plates at a density of 1 × 10⁴ cells/well and incubated for 20 h to allow for cell attachment. The culture medium was then replaced with fresh medium containing nanobodies at seven concentrations (3.125, 6.25, 12.5, 25, 50, 100, and 200 μM). Each concentration was tested in triplicate, and vehicle-treated cells served as the control.

Following 24 h of treatment, MTT solution was added to each well to a final concentration of 1 mg/mL, and the plates were incubated for an additional 5 h at 37°C. The supernatant was then carefully removed, and the resulting formazan crystals were dissolved in 100 μL of dimethyl sulfoxide (DMSO) with agitation for 10 min. The absorbance was measured at 450 nm using a Tecan Spark microplate reader. Relative cell viability (%) was calculated relative to the vehicle control group, and the half-maximal cytotoxic concentration (CC_50_) was determined using non-linear regression analysis in GraphPad Prism 6.

### Murine model of PLpro-induced colonic inflammation

Seven-week-old female C57BL/6J mice were utilized to evaluate the protective efficacy of the nanobodies against PLpro-induced inflammation. The mice were randomly assigned to the following experimental groups: normal control (*n =* 3), model control (*n =* 4), positive control (coptisine sulfate; *n =* 4), and nanobody treatment groups (2.5, 5, and 10 mg/kg; *n =* 4 per group).

A pre-treatment regimen was employed, consisting of daily intraperitoneal (i.p.) injections of TAT-conjugated nanobodies for three consecutive days. Following this period, intestinal inflammation was induced as previously described (27). At the study’s endpoint, the mice were euthanized, and colonic tissues, along with major organs (heart, liver, spleen, lungs, and kidneys), were harvested for subsequent histopathological evaluation and molecular analysis.

### *In vivo* efficacy of TAT nanobodies in K18-hACE2 neonatal mice

Neonatal K18-hACE2 transgenic mice (C57BL/6 background) were obtained from Jiangsu Jicui Yaokang Biological Technology Co., Ltd. (Nanjing, China). SARS-CoV-2 trVLPs were propagated in Caco2-Flag-mCherry-ACE2 cells, concentrated, and stored at −80°C. Viral titers were determined by TCID₅₀ assays as previously described (32). Neonatal mice were housed with their dams under a 12-h light/dark cycle throughout the study.

On postnatal day 0 (P0), mice were randomly assigned to experimental groups (*n* = 4 per group). Without anesthesia, mice were challenged intranasally (i.n.) with 1.0 × 10⁵ TCID₅₀ of SARS-CoV-2 trVLP in a total volume of 20 μL. Six hours post-infection, treatment was initiated. The experimental groups included: (i) Normal control; (ii) Model (trVLP only); (iii) TAT-NbP1 or TAT-NbP2 at three dose levels (2.5, 5, and 10 mg/kg); and (iv) Jun12682 (15 mg/kg) as a positive control (48). Nanobodies were administered intranasally on days 0, 2, 4, and 6. On day 7, mice were euthanized, and lung tissues were harvested. Samples were fixed in 4% paraformaldehyde overnight and processed for H&E staining to evaluate pathological changes.

### Real-time quantitative PCR

Total RNA was extracted from treated NCM460 cells using the Total RNA Extraction Kit (Epizyme, China) and from colonic tissues using TRIzol reagent (Invitrogen, USA) according to the manufacturers’ instructions. The concentration and purity of the isolated RNA were determined spectrophotometrically. Subsequently, the RNA was reverse-transcribed into cDNA using a reverse transcription master mix (Epizyme).

Quantitative PCR (qPCR) was performed using SYBR Green Master Mix (Epizyme) on a LightCycler 96 system (Roche, Mannheim, Germany). The reaction conditions were as follows: 95°C for 30 s, followed by 40 cycles of 95°C for 5 s and 60°C for 30 s. β-actin served as the internal reference gene for normalization. The primer sequences for all target genes are provided in Table S2 to S4. Relative mRNA expression levels were calculated using the 2^−ΔΔCt^ method.

### Epitope mapping via phage display

To identify the specific epitopes recognized by the nanobodies, a phage-displayed peptide library was constructed. Briefly, 60 overlapping peptides (20 amino acids in length with a 15-residue overlap) spanning the full-length SARS-CoV-2 PLpro sequence were synthesized and cloned into the pComb-3XSS vector (Synbio Technologies, Suzhou, China). The resulting plasmid library was introduced into *Escherichia coli* TG1-competent cells via electroporation. Phage particles were rescued and amplified using M13K07 helper phage, followed by purification and concentration via PEG/NaCl precipitation.

Biopanning was performed by immobilizing the target nanobodies onto 96-well plates. To minimize non-specific binding, the phage library was pre-incubated with 5% (wt/vol) skim milk in PBS. The blocked phages were then subjected to affinity selection against the immobilized nanobodies. Bound phages were eluted with trypsin and re-amplified in TG1 cells for subsequent rounds of selection. To increase selection stringency, the concentration of the coating nanobody was progressively reduced across five rounds (100, 50, 10, 5, and 2.5 μg/well). Following the final round of enrichment, positive clones were randomly selected for DNA sequencing. Specific binding epitopes were identified by aligning the enriched peptide sequences with the primary structure of the PLpro antigen.

### Statistical analysis

Statistical analyses were performed using GraphPad Prism 6.0 (GraphPad Software, San Diego, CA, USA). Data are presented as the mean ± standard deviation (SD). For comparisons between two groups, Student’s *t*-test was employed, while one-way analysis of variance (ANOVA) was used for comparisons involving three or more groups. All experiments were conducted with at least three independent replicates. A *P*-value < 0.05 was considered statistically significant.

## SUPPLEMENTAL MATERIAL

Supplemental material jvi.00599-26-s0001.docx Fig. S1 to S7; Tables S1 to S4.

ASM does not own the copyrights to Supplemental Material that may be linked to, or accessed through, an article. The authors have granted ASM a non-exclusive, world-wide license to publish the Supplemental Material files. Please contact the corresponding author directly for reuse.
