# Supplementary material for: Nanobodies targeting SARS-CoV-2 papain-like protease exert dual antiviral and anti-inflammatory effects
Source: J Virol. 2026 Aug 28;100(9):e00599-26. doi: 10.1128/jvi.00599-26 (PMC13595953; doi:10.1128/jvi.00599-26)
Supplement: Supplementary file 1 [file jvi.00599-26-s0001.docx]

**Supplemental information**

Nanobodies targeting SARS-CoV-2 papain-like protease exert dual antiviral and anti-inflammatory effects

Guolong Liu^a#^, Jiantao Chen^b,c#^, Fang Wu^a#^, Peilan Wei^b,c^, Yuzhe Wu^a^, Di Yan^a^, Xinfeng Xu^a^, Qiang Ding^d^, Yanqun Wang^b,c^, Shuwen Liu^a,e*^, Jincun Zhao^b,c*^, Wei Xu^a,e*^

^a^Guangdong Provincial Key Laboratory of New Drug Screening & NMPA Key Laboratory for Research and Evaluation of Drug Metabolism & Guangdong-Hong Kong-Macao Joint Laboratory for New Drug Screening, School of Pharmaceutical Sciences, Southern Medical University, Guangzhou, 510515, China.

^b^Guangdong Provincial Center for Disease Control and Prevention, Guangdong Provincial Key Laboratory of Pathogen Detection for Emerging Infectious Disease Response, Guangdong Workstation for Emerging Infectious Disease Control and Prevention, Chinese Academy of Medical Sciences, Guangzhou, People's Republic of China

^c^State Key Laboratory of Respiratory Disease, National Clinical Research Center for Respiratory Disease, Guangzhou Institute of Respiratory Health, the First Affiliated Hospital of Guangzhou Medical University, Guangzhou, Guangdong, 510180, China

^d^Center for Infection Biology, School of Basic Medical Sciences, Tsinghua University, Beijing 100084, China

^e^State Key Laboratory of Multi-organ Injury Prevention and Treatment, MOE Key Laboratory of Infectious Diseases Research in South China, MOE Innovation Center for Medical Basic Research on Inflammation and Immune Related Diseases, Southern Medical University, Guangzhou, 510515, China.

^*^E-mail addresses of corresponding authors: xuwei3322@smu.edu.cn (W.X), zhaojincun@gird.cn (J.Z), liusw@smu.edu.cn (S. L)

**Figure S1**

Figure S1. Binding kinetics and specificity of nanobody CW056 for PLpro. SPR sensorgrams illustrating the interaction between nanobody CW056 and recombinant PLpro. The binding profiles were analyzed using a 3D-printed microfluidic biosensor chip.

**Figure S2**

Figure S2. Inhibition of PLpro proteolytic activity by TAT-conjugated nanobodies.

(A) Schematic representation of the in vitro biochemical assay protocol used to evaluate the inhibitory potential of nanobodies against PLpro.

(B-C) Dose-response curves illustrating the dose-dependent inhibition of PLpro proteolytic activity by (B) TAT-NbP1 and (C) TAT-NbP2. Data points represent the mean ± SD of three independent experiments.

**Figure S3**

Figure S3. Immunofluorescence analysis of nanobody localization in Caco2-N cells. Representative immunofluorescence images showing Caco2-N cells treated with 1 μM of NbP1, NbP2, or TAT-CW056. TAT-CW056 (targeting Ebola VP30) was used as a non-binding negative control. Images are representative of three independent experiments (*n=3*).

**Figure S4**

Figure S4. Antiviral activity of chloroquine and negative control nanobody TAT-CW056 against SARS-CoV-2 variants.

(A) Representative immunofluorescence images of Vero E6 cells treated with serial dilutions of chloroquine (0.01–10 μM) or the negative control nanobody TAT-CW056 (30 μM, anti-Ebola VP30) and infected with SARS-CoV-2 (WT or EG.5 strains). Images are representative of three independent experiments (*n=3*).

(B-C) Dose-response curves illustrating the infection rates for (B) SARS-CoV-2 WT and (C) the EG.5 variant. Infection rates were quantified based on fluorescence intensity using ImageJ software. Data are presented as mean ± SD (*n=3*).

**Figure S5**

Figure S5. Interaction of PLpro with ISG15 and inhibition of trVLP replication in NCM460 cells.

(A) Fluorescence polarization (FP) binding curve showing the interaction between recombinant PLpro and FITC-labeled ISG15.

(B) Quantification of GFP expression levels in NCM460 cells following infection with SARS-CoV-2 trVLP and subsequent treatment with the indicated nanobodies. GFP serves as a reporter for viral replication/transcription. Data represent mean ± SD (*n=3*).

**Figure S6**

Figure S6. Body weight changes in mice across experimental groups.

**Figure S7**

Figure S7. Cytotoxicity of TAT-fused nanobodies in NCM460 cells.

(A) Cell cytotoxicity results of NbP1 before and after TAT fusion in NCM460 cells.

(B) Cell cytotoxicity results of NbP2 before and after TAT fusion in NCM460 cells.

**Table S1. The primers for TAT fusion sequencing.**

| Name | Primer sequence (5'-3') |
| --- | --- |
| WX1091 | GCGACGGCGCTGGCGGCGTTTTTTGCGGCCATAGTGGTGGTGGTGGTGGTGCTCG |
| WX1092 | TATGGCCGCAAAAAACGCCGCCAGCGCCGTCGCTGAGATCCGGCTGCTAACAAAG |
| WX1093 | GGGGGTAATGATACCGATGAAACGAGAGAG |
| WX1094 | CTCTCTCGTTTCATCGGTATCATTACCCCC |
| WX1095 | GCGACGGCGCTGGCGGCGTTTTTTGCGGCCATAGCCCATGGCCATCGCCGGCTGG |
| WX1096 | TATGGCCGCAAAAAACGCCGCCAGCGCCGTCGCCAGGTGCAGCTGCAGGAAAGCG |

**Table S2. The Primers of qRT–PCR (NCM460 cell line).**

| Genes Name | Primer sequence (5'-3'), Forward | Primer sequence (5'-3'), Reverse |
| --- | --- | --- |
| hTNF-α | TGGCCCAGACCCTCACACTCAG | ACCCATCGGCTGGCACCACT |
| hIL-1α | CGAAGACTACAGTTCTGCCAT | ATATGTGATGCCCTGGTGGT |
| hISG15 | GAGGCAGCGAACTCATCTTT | AGCATCTTCACCGTCAGGTC |
| hβ-actin | GGCACCCAGCACAATGAAG | CCGATCCACACGGAGTACTTG |

**Table S3. The Primers of qRT–PCR sequencing (Animal experiments).**

| Genes Name | Primer sequence (5'-3'), Forward | Primer sequence (5'-3'), Reverse |
| --- | --- | --- |
| mTNF-α | CCACCACGCTCTTCTGTCTAC | AGGGTCTGGGCCATAGAACT |
| mIL-1α | AGCTTGACGGCACCCTCGCA | CGGAGAGCTTCGTGGCTGTGGA |
| mISG15 | GGTGTCCGTGACTAACTCCAT | TGGAAAGGGTAAGACCGTCCT |
| mβ-actin | CCTCTATGCCAACACAGTGC | CCTGCTTGCTGATCCACATC |

**Table S4. Primers required for the detection of lung-related biochemical markers in K18-hACE2 mice.**

| Genes Name | Primer sequence (5'-3'), Forward | Primer sequence (5'-3'), Reverse |
| --- | --- | --- |
| mTNF-α | CCACCACGCTCTTCTGTCTAC | AGGGTCTGGGCCATAGAACT |
| mIL-1β | AAGCCCTTGCTGTAGTGGTG | GAAGCTGATGGCCCTAAACA |
| mISG15 | GGTGTCCGTGACTAACTCCAT | TGGAAAGGGTAAGACCGTCCT |
| Spike | GCTGGTGCTGCAGCTTATTAT | AGGGTCAAGTGCACAGTCTA |
| GFP | AAGTTCATCTGCACCACCG | TCCTTGAAGAAGATGGTGCG |
